# KCC2-dependent subcellular E_Cl_ difference of ON-OFF retinal ganglion cells in larval zebrafish

**DOI:** 10.3389/fncir.2013.00103

**Published:** 2013-05-28

**Authors:** Rong-wei Zhang, Shu-yi Zhang, Jiu-lin Du

**Affiliations:** Institute of Neuroscience and State Key Laboratory of Neuroscience, Shanghai Institutes for Biological Sciences, Chinese Academy of SciencesShanghai, China

**Keywords:** Cl^−^ reversal potential, GABA, KCC2, subcellular, retinal ganglion cells, *in vivo* whole-cell recording, zebrafish

## Abstract

Subcellular difference in the reversal potential of Cl^−^ (E_Cl_) has been found in many types of neurons. As local E_Cl_ largely determines the action of nearby GABAergic/glycinergic synapses, subcellular E_Cl_ difference can effectively regulate neuronal computation. The ON-OFF retinal ganglion cell (RGC) processes both ON and OFF visual signals via its ON and OFF dendrites, respectively. It is thus interesting to investigate whether the ON and OFF dendrites of single RGCs exhibit different local E_Cl_. Here, using *in vivo* gramicidin-perforated patch recording in larval zebrafish ON-OFF RGCs, we examine local E_Cl_ at the ON and OFF dendrites, and soma through measuring light-evoked ON and OFF inhibitory responses, and GABA-induced response at the soma, respectively. We find there are subcellular E_Cl_ differences between the soma and dendrite, as well as between the ON and OFF dendrites of single RGCs. These somato-dendritic and inter-dendritic E_Cl_ differences are dependent on the Cl^−^ extruder, K^+^/Cl^−^ co-transporter (KCC2), because they are largely diminished by down-regulating *kcc2* expression with morpholino oligonucleotides (MOs) or by blocking KCC2 function with furosemide. Thus, our findings indicate that there exists KCC2-dependent E_Cl_ difference between the ON and OFF dendrites of individual ON-OFF RGCs that may differentially affect visual processing in the ON and OFF pathways.

## Introduction

Intracellular Cl^−^ homeostasis is involved in the regulation of many cellular functions, including cell volume and membrane excitability (Blaesse et al., 2009). In the neurons of neonatal brains, developmental up-regulation of the Cl^−^ extruder K^+^/Cl^−^ co-transporter (KCC2) and down-regulation of the Cl^−^ importer Na^+^/K^+^/Cl^−^ (NKCC) cause progressive reduction of intracellular chloride concentration ([Cl^−^]_i_), resulting in a hyperpolarization shift of the Cl^−^ reversal potential (E_Cl_) and a switch of gamma aminobutyric acid (GABA) action from excitation to inhibition (Wang and Kriegstein, 2009; Ben-Ari et al., 2012).

In individual neurons, differential subcellular distribution of KCC2 and/or NKCC can generate an uneven [Cl^−^]_i_ gradient along neuronal processes, leading to different GABA actions on the same neuron (Vardi et al., 2000; Khirug et al., 2005; Duebel et al., 2006; Gavrikov et al., 2006). In starburst amacrine cells of rabbit retinae, KCC2 and NKCC2 are preferentially located at distal and proximal dendrites, respectively, resulting in GABA-evoked hyperpolarization at distal dendrites and depolarization at proximal dendrites. This inter-dendritic difference in the GABA action contributes to direction-selective light responses of those cells (Gavrikov et al., 2003, 2006). Similarly, the dendrite and axon of ON bipolar cells preferentially express NKCC and KCC2, respectively (Vardi et al., 2000; Duebel et al., 2006), resulting in a depolarization action of synaptic inputs from horizontal cells to BC dendrites and a hyperpolarization action of synaptic inputs from amacrine cells to BC axons. Therefore, non-uniform subcellular distribution of KCC2 and/or NKCC in retinal cells can regulate visual information processing.

The ON-OFF retinal ganglion cell (RGC) extends multi-stratified dendrites into both the sublamina *a* and *b* of the inner plexiform layer, where it receives visual information from OFF and ON bipolar cells, respectively. Moreover, both light-evoked ON and OFF responses of those cells can be modulated by inhibitory synaptic inputs originated from GABAergic and glycinergic amacrine cells. It is thus of interest to examine whether the action of GABAergic/glycinergic inhibition is different between the ON and OFF dendrites of ON-OFF RGCs. To address this question, we performed gramicidin-perforated patch recording in intact larval zebrafish, and examined Cl^−^ reversal potential (E_Cl_) at the soma, and ON and OFF dendrites of ON-OFF RGCs during 2.5–6 days post-fertilization (dpf). We found that there are subcellular E_Cl_ differences between the soma and dendrite (somato-dendritic), and between the ON and OFF dendrites (inter-dendritic). The E_Cl_ difference is largely dependent on KCC2 function because it was diminished by genetic knockdown or pharmacological blockade of KCC2.

## Materials and methods

### Zebrafish preparation

Wild-type AB adult zebrafish (*Danio rerio*) were maintained in the National Zebrafish Resources of China (Shanghai, China) with an automatic fish-housing system (ESEN, China) at 28°C. Embryos and larvae were raised on a 14–10 h light-dark cycle in 10% Hank's solution, which consists of (in mM) 140 NaCl, 5.4 KCl, 0.25 Na_2_HPO_4_, 0.44 KH_2_PO_4_, 1.3 CaCl_2_, 1.0 MgSO_4_, and 4.2 NaHCO_3_ (pH 7.2) (Westerfield, 1995). Electrophysiological recordings were performed on 2.5- to 6-dpf larval zebrafish at room temperature (22–26°C). All zebrafish handling procedures followed the guideline of Institute of Neuroscience, Chinese Academy of Sciences.

### Electrophysiological recording

**Figure 1:**
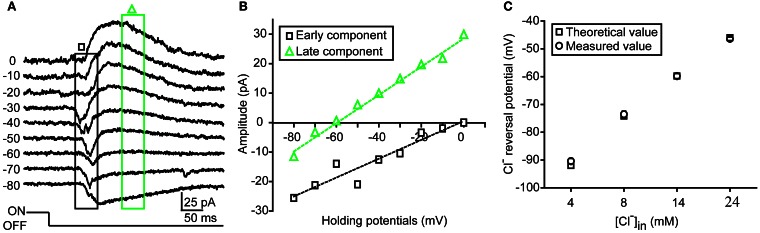
**Measurement of dendritic E_Cl_ in zebrafish RGCs. (A)** An example showing light-evoked OFF synaptic responses recorded at different holding potentials from −80 to 0 mV when the ON-OFF RGC was loaded with 14 mM Cl^−^ internal solution under conventional whole-cell recording mode. **(B)** I–V curves of early (black square) and late (green triangle) components of light-evoked responses. Dendritic E_Cl_, calculated by fitting the I–V curve of the late-phase component with linear regression, is about −60 mV, close to the Cl^−^ equilibrium potential (−59.8 mV). **(C)** Theoretical (square) and measured (circle) dendritic E_Cl_ when the RGCs was loaded with internal solution with different Cl^−^ concentration (4, 8, 14, or 24 mM). The data were obtained from 10 cells at each data point. The values are represented as mean ± SEM.

**Figure 2:**
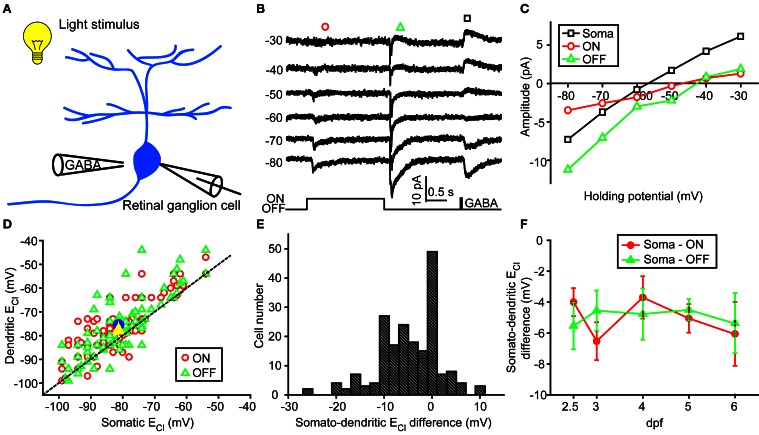
**Somato-dendritic E_Cl_ difference of zebrafish ON-OFF RGCs. (A)** A schematic showing the experimental procedure. **(B)** Light-evoked ON (red circle) and OFF (green triangle) synaptic responses and GABA-induced currents (black square) were simultaneously recorded at different holding potentials from an ON-OFF RGC under gramicidin-perforated patch recording mode. **(C)** I–V curves of GABA-induced current at the RGC soma (black square), and the late-phase components of ON (red circle) and OFF (green triangle) light-evoked responses. The data were obtained from the same cell with **(B)**. **(D)** Plots of dendritic E_Cl_ against somatic E_Cl_. The dotted line represents the orthogonal, and the blue circle and yellow triangle represent the mean E_Cl_ of ON and OFF responses, respectively. The data were obtained from 98 ON-OFF RGCs. **(E)** Distribution of somato-dendritic E_Cl_ difference. **(F)** Mean E_Cl_ differences between soma and ON or OFF dendrites of ON-OFF RGCs from 2.5 to 6 dpf. At each data points, the cell number is more than 13. The values are represented as mean ± SEM.

**Figure 3:**
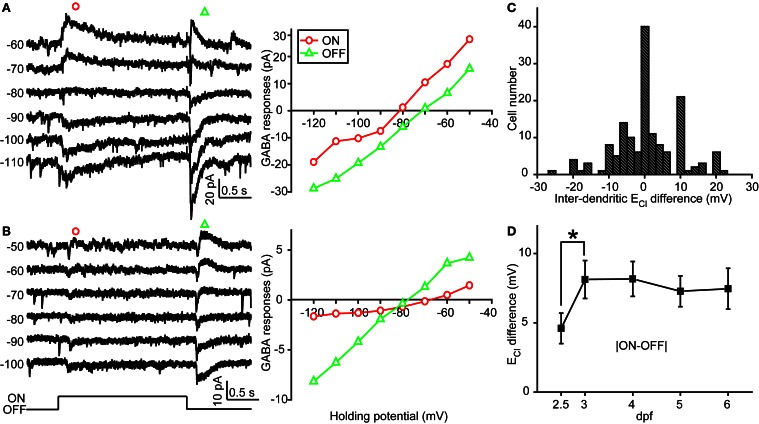
**Inter-dendritic E_Cl_ difference between ON and OFF dendrites of zebrafish ON-OFF RGCs. (A,B)** Left, light-evoked ON and OFF synaptic responses recorded at different holding potentials from two ON-OFF RGCs under gramicidin-perforated patch recording mode. Right, I–V curves of the late-phase components of light-evoked ON (red circle) and OFF (green triangle) responses. **(C)** Distribution of the inter-dendritic E_Cl_ difference from 152 cells. **(D)** Mean absolute values of inter-dendritic E_Cl_ difference from 2.5 to 6 dpf. At each data points, the cell number is more than 15. ^*^P < 0.05, unpaired Student's *t*-test. The values are represented as mean ± SEM.

**Figure 4:**
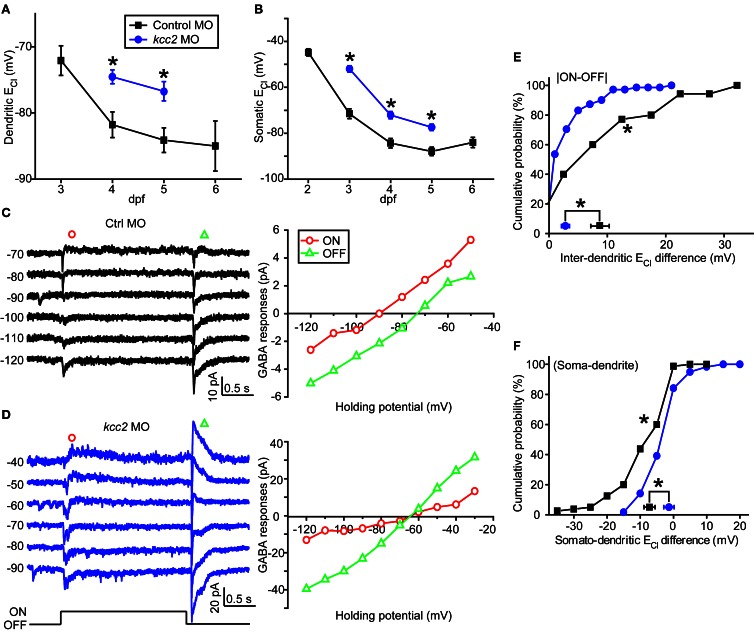
**Knockdown of zebrafish *kcc2* diminishes subcellular E_Cl_ difference. (A,B)** Mean dendritic **(A)** and somatic **(B)** E_Cl_ of control (black square) and *kcc2* morphants (blue circle). At each data points, the cell number is more than 8. **(C,D)** Left, Light-evoked ON and OFF responses of RGCs from a control **(C)** and *kcc2* morphants **(D)** under gramicidin-perforated patch recording mode. Right, I–V curves of the late-phase components of light responses. **(E,F)** Cumulative probability of inter-dendritic **(E)** and somato-dendritic **(F)** E_Cl_ differences in control (*n* = 35 cells) and *kcc2* morphants (*n* = 71 cells). The symbols near the X-axis represent the mean E_Cl_ differences. ^*^P < 0.01, Kolmogorov–Smirnov test. The values are represented as mean ± SEM.

**Figure 5:**
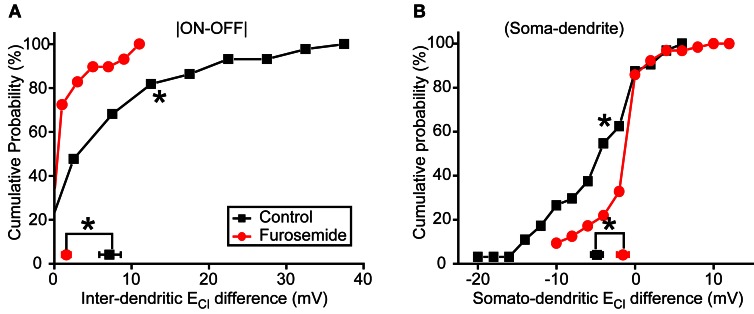
**Blockade of KCC2 reduces subcellular E_Cl_ difference. (A,B)** Cumulative probability of inter-dendritic **(A)** and somato-dendritic **(B)** E_Cl_ difference in control (*n* = 44 cells) and furosemide-treated (*n* = 29 cells) animals. The symbols near the X-axis represent the mean E_Cl_ differences. ^*^P < 0.01, Kolmogorov–Smirnov test. The values are represented as mean ± SEM.

*In vivo* whole-cell recordings were made from the cells at the ganglion cell layer of the retina according to our previous experimental procedure (Zhang et al., 2010). Based on previous reports (Kay et al., 2001; Wei et al., 2012), displaced amacrine cells are rarely observed in zebrafish larvae and the majority of cells at the ganglion cell layer are RGCs. After dissection, the larval preparation was continuously perfused with external solution, which consists of (in mM) 134 NaCl, 2.9 KCl, 2.1 CaCl_2_, 1.2 MgCl_2_, 10 HEPES and 10 glucose (290 mOsm/L, pH 7.8). Recording micropipettes were made from borosilicate capillaries (BF 120-69-15, Sutter Instrument), and had a resistance in the range of 10–15 MΩ. In order to measure physiological E_Cl_ of zebrafish RGCs, we performed gramicidin-perforated patch recording, with which intracellular Cl^−^ homeostasis was not disturbed (Kyrozis and Reichling, 1995). The pipette was tip-filled with gramicidin-free internal solution and then back-filled with internal solution containing 10 μg/ml gramicidin. For gramicidin recordings (Figures 2–5), we used internal solution with high Cl^−^ concentration that contains (in mM) 110 KCl, 6 NaCl, 2 CaCl_2_, 2 MgCl_2_, 10 HEPES, and 10 EGTA (260 mOsm/L, pH 7.4). For calibrating dendritic E_Cl_ measurement (Figure 1), we performed conventional whole-cell recordings with low Cl^−^ concentration-containing internal solution. The recording was made with an EPC-10 amplifier (Heka, Germany), and signals were filtered at 2.9 kHz and sampled at 10 kHz. Data were discarded if the series resistance varied >20% during recordings. To evoke light responses of RGCs, 2-s whole-field light flash was given via a cooled light source controlled by an electrical shutter (LS6Z2, Uniblitz). All drugs were purchased from Sigma (St. Louis).

### Knockdown of zebrafish *kcc2*

Morpholino oligonucleotides (MOs) were used to down-regulate the expression of zebrafish *kcc2* gene. The *kcc2* MO (TGGATGTTGCATCTCCTGTGAACAT) and a standard control MO (CCTCCTACCTCAGTTACAATTTATA) were produced by Gene Tools (Philomath, OR), and used in our previous study (Zhang et al., 2010). A dose of 8-ng MO was pressure-injected into the animal pole of zebrafish embryos at 1-cell stage. Since the MOs were tagged with the fluorophore lissamine at the 3′ terminal, the distribution of MOs in zebrafish RGCs could be visualized by fluorescent signal.

### Statistical analysis

Lillie test was first performed to examine the normality assumption of data. For normal data, paired or unpaired student's *t*-test was used for statistical significance analysis between two groups. For data which was not normal, Wilcoxen sign-rank test was then used for significance analysis. For testing the significance of the differences in the cumulative distribution of E_Cl_, the non-parametric Kolmogorov–Smirnov test was used (Figures 4, 5). The *P*-value <0.05 was considered to be statistically significant. All results were represented as mean ± SEM.

## Results

### Measurement of RGC dendritic E_CL_

Under infra-red visual guidance, RGCs in intact zebrafish larvae aged from 2.5 to 6 dpf were recorded, and their light responses were evoked by the application of 2-s whole-field light flashes. In the present study, ON-OFF RGCs, which responded to both the onset and offset of light stimuli, were selected for investigation. Most of those cells exhibited small bi-stratified or diffuse dendritic fields (Zhang et al., 2010). Similar to other species (Gao and Wu, 1998; Pang et al., 2003), light-evoked responses (LERs) of zebrafish ON-OFF RGCs could be temporally divided into two synaptic components after the onset of the responses (Figure 1). In order to confirm the measurement of dendritic E_Cl_ based on light-evoked inhibitory component, we firstly performed conventional whole-cell recording on RGC soma with internal solution containing low Cl^−^ concentration (14 mM) (Figures 1A,B). The early component of OFF LER (0–50 ms after the LER onset) was reversed at ~0 mV (Figures 1A,B), which is believed to be mediated by glutamatergic inputs from bipolar cells. Meanwhile, the late component (150–200 ms after the LER onset) was reversed around −60 mV, similar to the theoretical E_Cl_ according to the Nernst equation (Figure 1B), supporting the notion that this component was mediated by GABAergic/glycinergic inputs from amacrine cells.

As inhibitory synapses are mainly formed at RGC dendrites, the reversal potential of the late-phase ON and OFF light responses can reflect the local E_Cl_ of ON and OFF dendrites of RGCs, respectively. To further confirm this point, we loaded ON-OFF RGCs with internal solution containing different Cl^−^ concentration (4, 8, 14, or 24 mM) under conventional whole-cell recording mode. We found that the average reversal potential of late-phase LERs was almost the same as the theoretical E_Cl_: at 4 mM, −90.4 ± 1.0 vs. −92.0 mV; at 8 mM, −73.6 ± 1.1 vs. −74.2 mV; at 14 mM, −59.8 ± 0.9 vs. −59.8 mV; at 24 mM, −46.4 ± 0.6 vs −46 mV (Figure 1C).

### Somato-dendritic and inter-dendritic E_CL_ differences

In order to examine physiological E_Cl_ of ON-OFF RGCs, we performed gramicidin-perforated recordings in all following experiments. Besides dendritic E_Cl_ measurement based on light-evoked inhibitory responses, we also examined somatic E_Cl_ through puffing GABA solution (50 μM) onto RGC soma via a glass micropipette (Figure 2A) (Zhang et al., 2010). The peak amplitude of GABA-induced currents was measured when the ON-OFF RGC was held at different potentials (square, Figure 2B). Interestingly, there were obvious differences between somatic and dendritic E_Cl_ in single RGCs (Figures 2B,C). Somatic E_Cl_ was more negative than both ON and OFF dendritic E_Cl_ (soma vs. ON dendrite, −81.3 ± 1.1 vs. −76.1 ± 1.1 mV, *n* = 98, *P* < 0.0001; soma vs. OFF dendrite, −81.3 ± 1.1 vs. −77.1 ± 1.3 mV, *n* = 98, *P* < 0.0001, paired student's *t*-test; Figure 2D), and the somato-dendritic E_Cl_ difference varied in individual cells (Figure 2E). During 2.5–6 dpf, the average E_Cl_ difference between the soma and ON or OFF dendrite remained relatively consistent (Figure 2F). Furthermore, there was also a difference between ON and OFF dendritic E_Cl_ in individual RGCs (Figure 3). In 53 out of 152 ON-OFF RGCs, ON dendritic E_Cl_ was more negative than OFF dendrite E_Cl_ (Figures 3A,C), whereas the situation was reversal in other 60 cells (Figures 3B,C). In total, the absolute difference between ON and OFF dendritic E_Cl_ ranged from 4.6 to 8.2 mV during 2.5–6 dpf (Figure 3D), and exhibited a significant increase from 2.5 to 3 dpf (*P* < 0.05), implying a developmental change of subcellular Cl^−^ homeostasis between ON and OFF dendrites during this stage. Taken together, there is a Cl^−^ gradient within subcellular compartments of larval zebrafish ON-OFF RGCs.

### Kcc2 regulates subcellular E_CL_ differences

Since intracellular Cl^−^ homeostasis is maintained by the action of cation/Cl^−^ co-transporters (CCCs) (Blaesse et al., 2009), the difference between somatic and dendritic E_Cl_ in zebrafish ON-OFF RGCs implies differential distribution of CCCs, e.g., KCC2, at different subcellular regions of these cells. To examine this point, we micro-injected morpholino oligos (MOs) into zebrafish embryos at 1-cell stage to down-regulate *kcc2* expression (see Materials and Methods). Knockdown of zebrafish *kcc2* resulted in a shift of both the dendritic and somatic E_Cl_ in ON-OFF RGCs toward a depolarization level (Figures 4A,B), indicating the effectiveness of *kcc2* MO (Zhang et al., 2010). Importantly, the absolute value of inter-dendritic E_Cl_ difference between ON and OFF dendrites of RGCs was significantly reduced in *kcc2* morphants in comparison to the control group (*kcc2* MO, 2.8 ± 0.9 mV; control MO, 8.7 ± 1.6 mV; *P* < 0.01, Kolmogorov–Smirnov test; Figures 4C–E). Similar reduction was observed in somato-dendritic E_Cl_ difference (*kcc2* MO, −1.8 ± 0.9 mV; control MO, −7.4 ± 1.3 mV; *P* < 0.01, Kolmogorov–Smirnov test; Figure 4F).

To further confirm the role of KCC2 in the subcellular difference of E_Cl_, we then applied furosemide (50 μM), a broad-spectrum inhibitor of CCCs (Payne et al., 2003), to suppress KCC2 function. Similar to KCC2 knockdown experiments, bath application of furosemide significantly reduced inter-dendritic E_Cl_ difference from 7.2 ± 1.4 to 1.6 ± 0.6 mV (*P* < 0.01, Kolmogorov–Smirnov test; Figure 5A) and somato-dendritic E_Cl_ difference from −4.8 ± 0.8 to −1.5 ± 0.5 mV (*P* < 0.01, Kolmogorov–Smirnov test; Figure 5B). Taken together, these results indicate that KCC2 plays an important role in the establishment of the subcellular difference of E_Cl_ in zebrafish ON-OFF RGCs.

## Discussion

**Figure 6:**
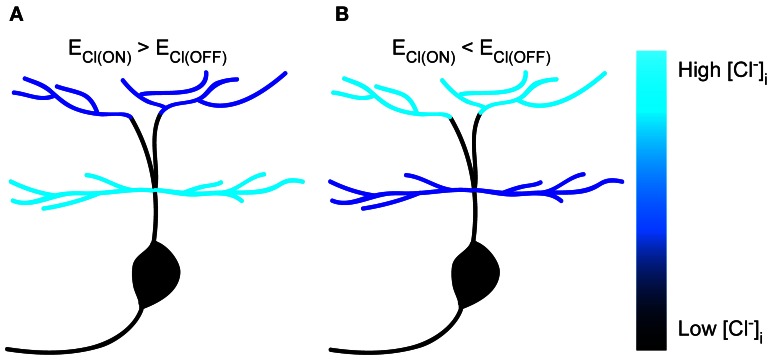
**Schematic of subcellular E_Cl_ differences within single zebrafish RGCs. (A,B)** Cartoon showing subcellular E_Cl_ differences between soma, ON and OFF dendrites in zebrafish ON-OFF RGCs. The [Cl^−^]_i_ is color-coded.

In the present study, we performed *in vivo* gramicidin-perforated recordings in developing zebrafish ON-OFF RGCs, and found somato-dendritic and inter-dendritic E_Cl_ differences (Figure 6), which were largely dependent on KCC2 function. Our findings indicate that KCC2 plays an important role in the establishment of subcellular E_Cl_ differences in RGCs.

Spatial distribution of KCC2 and/or NKCC1 produces subcellular [Cl^−^]_i_ gradient along different parts of individual neurons. In hippocampal principal cells, KCC2 is primarily expressed at dendritic spines, but rarely at soma and dendritic shafts (Gulyas et al., 2001), causing a somato-dendritic [Cl^−^]_i_ gradient (Khirug et al., 2005). In the retina, KCC2 and NKCC1 are preferentially expressed at the axon terminals and dendrites of ON bipolar cells, respectively (Vardi et al., 2000), implying the existence of subcellular [Cl^−^]_i_ gradient in bipolar cells. Consistently, it is found that the soma of ON bipolar cells exhibits more negative E_Cl_ than their dendrites (Duebel et al., 2006). Therefore, ON bipolar cells may receive depolarizing GABAergic actions from horizontal cells at their dendrites, but receive hyperpolarizing GABAergic inputs from amacrine cells at their axon terminals.

In zebrafish larvae, the expression of *kcc2* is first detected at 2 dpf, and increased in following days (Reynolds et al., 2008), leading to an excitation-to-inhibition switch of GABAergic action in RGCs at 2.5 dpf (Zhang et al., 2010). Our data show that somato-dendritic and inter-dendritic [Cl^−^]_i_ gradients already exist even at 2.5 dpf (Figures 2F, 3D), a time point when zebrafish RGCs begin to exhibit LERs (Zhang et al., 2010). This suggests that the initial establishment of subcellular [Cl^−^]_i_ gradient is intrinsic and not dependent on visual experience. Notably, the increase of inter-dendritic E_Cl_ difference occurs between 2.5 and 3 dpf, time points when the level of *kcc2* expression is elevated (Reynolds et al., 2008; Zhang et al., 2010). Moreover, our findings that knockdown of *kcc2* expression or pharmacological blockade of KCC2 function largely diminished inter-dendritic [Cl^−^]_i_ gradients imply differential KCC2 distribution between ON and OFF dendrites, perhaps due to sequential formation of RGC dendrites. Mumm and colleagues performed *in vivo* time-lapse imaging in developing zebrafish RGCs, and found that the inner dendritic arbor of some RGCs first formed, following by the addition of dendritic strata in the outer sublamina of the inner plexiform layer, *vice versa* in other RGCs examined (Mumm et al., 2006). This sequential growth of multi-stratified dendrites may cause differential KCC2 expression between ON and OFF dendrites, leading to inter-dendritic E_Cl_ difference in developing zebrafish RGCs. However, due to the lack of specific antibodies for zebrafish KCC2, we cannot examine the subcellular distribution of KCC2 within an individual RGC. Despite the lack of *kcc2* zebrafish antibodies, a genetic approach could be used. For example, generating a transgenic line expressing *kcc2*-GFP and/or NKCC1-RFP will be helpful for us to correlate electrophysiology directly with the *kcc2*/NKCC1 ratio at different subcellular compartments. Furthermore, we could combine voltage-sensitive and chloride-sensitive dyes to confirm whether different *kcc2*/NKCC1 ratios may change ECl at different cellular regions, leading to differential GABA-induced actions. In the future, KCC2 immunostaining and intracellular Cl^−^ imaging in zebrafish RGCs may help us understand the mechanism underlying the formation of subcellular [Cl^−^]_i_ gradient.

As local E_Cl_ can determine the strength or even polarity of GABAergic/glycinergic synaptic inputs, the inter-dendritic E_Cl_ difference in ON-OFF RGCs may lead to a differential extent of inhibition at ON and OFF dendrites, and cause asymmetric light-evoked spiking activity between ON and OFF visual pathways (Joselevitch and Kamermans, 2009; Zhang and McCall, 2012). Therefore, subcellular E_Cl_ differences in zebrafish RGCs can play a role in visual information processing.

### Conflict of interest statement

The authors declare that the research was conducted in the absence of any commercial or financial relationships that could be construed as a potential conflict of interest.
